# Wake Characteristics of Multiscale Buildings in a Turbulent Boundary Layer

**DOI:** 10.1007/s10546-025-00910-3

**Published:** 2025-05-05

**Authors:** Cameron Southgate-Ash, Abhishek Mishra, Sue Grimmond, Alan Robins, Marco Placidi

**Affiliations:** 1 Department of Mechanical Engineering Sciences, Environmental Flow Research Centre (EnFlo), University of Surrey, Guildford, UK; 2 Department of Meteorology, University of Reading, Reading, UK

**Keywords:** Drag measurements, Multi-scale roughness, Turbulent boundary layer, Wake flows, Wind tunnel experiments, Fractal buildings

## Abstract

Urban forms characterised by multi-scale roughness can drastically modify the wind structure within cities affecting both pedestrian comfort and air quality at street level. For simplicity, most urban flow studies focus on cuboid buildings with a single length scale. We consider six forms to assess how additional length scales impact urban flow: two reference cuboids that differ in aspect ratio (mean building height to width) cases (Standard, 1; Tall, 3) plus two additional fractal iterations of each. The six models have the same mean building width, height, and frontal area but their length scale characteristics differ. These are used in wind tunnel experiments within a deep turbulent boundary layer. The length scale differences are found to affect the drag force exerted by the buildings in a non-negligible way (up to 5 and 13% for Standard and Tall buildings, respectively). The added length scales also modify the wake lateral spread and intensity of the turbulence fluctuations, with smaller the length scales having the lower (higher) intensity of fluctuations in the near (far) wake. Additionally, the strength of the vortex shedding emanating from the buildings is reduced by introducing systematically smaller length scales. This work suggests that omission of additional length scales can lead to inaccuracies in drag and wake recovery estimations. The reduction in intensity of vortex shedding found with each fractal iteration could have engineering applications (e.g. reducing vibration).

## Introduction

By 2050 it is estimated that cities, where tall buildings predominate, will be home to more than two-thirds of the world’s population (DESA 2019). Therefore, it is increasingly important to understand the flow in urban environments, particularly the contribution of tall buildings. Tall buildings are those with an aspect ratio (AR or mean height to width) of $3<AR<8$, as defined by the Council on Tall Buildings and Urban Habitat (2019). For simplicity, buildings are typically modelled (experimentally or numerically) with simple square or rectangular cylinders, which are a classical bluff body problem (Bearman and Obasaju 1982; Lyn and Rodi 1994; Voke 1997; Minguez et al. 2011; Trias et al. 2015; Daniels et al. 2016). A standardised simplified tall building model named ‘CAARC’ is also often considered in wind engineering as a case study in wind loading (Melbourne 1980; Alexandre and Armando 2009; Alminhana et al. 2018; Hertwig et al. 2019). These simplifications extend to most urban flow studies that focus on surfaces covered with cuboid buildings with a single length scale (Cheng and Castro 2002; Coceal et al. 2006; Castro 2007; Leonardi and Castro 2010; Castro et al. 2017). Although cuboids are an effective representation of some simplified urban morphologies, they are often an oversimplification when compared to real urban data (Grimmond and Oke 1999; Barlow and Coceal 2009; Makedonas et al. 2021). Most buildings, in particular tall buildings, in fact, are multi-scale (i.e. characterised by several length scales), such as height being several times larger than width, and width being an order of magnitude larger than facade features, such as balconies or other architectural features (Saroglou et al. 2017).

To date, multi-scale roughness work is still underrepresented in the literature. Vanderwel and Ganapathisubramani (2019) show that the smallest geometric scales (those an order of magnitude smaller than the largest) have negligible impact on the overall drag generated by a rough surface. Multiscale urban morphologies have also been explored through heterogeneity in building height (Pascheke et al. 2008; Kanda et al. 2013; Makedonas et al. 2021; Kim et al. 2023). These works have highlighted how additional scales through variation in building height can significantly enhance turbulent fluctuations and mixing. Furthermore, Kanda et al. (2013) shows that the inclusion of heterogeneity in aerodynamic parametrisation yields an improved estimation of the roughness length and displacement height over real urban morphologies when compared to conventional methods. Studies investigating the effect of additional length scales added to building models themselves are also not common in the literature. However, some works have explored this aspect when looking at the effect of different levels of detail characterising the models (e.g. roof shapes in Garau et al. (2018); Coburn et al. (2021)) on the overall flow field. Garau et al. (2018) demonstrated that the inclusion of a gable roof, compared to a flat roof, increases momentum flux and promotes higher ventilation, particularly in narrow street canyons. Moreover, Coburn et al. (2021) showed that pitched roofs can lead to significant changes in the mean flow field, Reynolds stresses and aerodynamics drag generated by the canopy. Multi-scale vegetation canopies (i.e. inherently fractal trees) research also provided insights into flow structure within the canopy layer, and the link between fractal length scale and dispersive stresses behaviour. Bai et al. (2015) observed that the mixing length decreases with height within the canopy, which contradicts the classic boundary layer theory. These finding stress the importance of including multi-scale information in urban flow modelling.

A convenient methodology to implement a systematic introduction of multi-scale information is by using fractal geometries. Nedić et al. (2013) showed that for isolated fractal flat plates, the smallest scales can have a non-negligible (up to 7%) impact on drag. Wake size was also found to have some dependence on the smaller scales, with an increase in fractal dimension leading to a decrease in wake size. The higher iteration fractal plates (i.e. smallest length scales) also show less turbulence intensity in the near-wake region, recovering more slowly than base iteration plates with more turbulence activity downstream (i.e. a more persistent wake). The shedding frequency was observed to remain the same with an increase in fractal dimension, but the intensity of the shedding decreased. Despite the Nedić et al. (2013) plates not being submerged in a boundary layer, clear changes in drag and wake characteristics were seen without changing the bluff body frontal area. This finding - if extended to a full surface comprising of fractal objects - would be in disagreement with customary rough wall modelling where many morphometric methods prescribe the roughness length and zero-plane displacement to depend purely on frontal/plan areas (Bottema 1993; Macdonald et al. 1998). Indeed, Medjnoun et al. (2021)’s study of four multi-scale surfaces also found that the smallest scales contribute to 7% of the overall drag. More widely, many have explored turbulence generated by multi-scale fractal grids (Hurst and Vassilicos 2007; Valente and Vassilicos 2011; Thormann and Meneveau 2014; Vassilicos 2015, and references therein). While these provide valuable insights into the validity of canonical turbulence scaling and decay, they have a tenuous link to our focus on urban forms, so we do not discuss this topic any further.

Finally, a note on the terminology in use herein. We employ the term ‘roughness’ following classical boundary layer theory to signify a downward shift of the log-law portion of the velocity profiles characterising turbulent boundary layers. i.e. akin to roughness length, $z_0$ (Pope 2000). Therefore, we note that most prior multi-scale roughness work focuses on single-scale roughness elements arranged in multi-scale patterns (Yang and Meneveau 2017; Zhu et al. 2017; Vanderwel and Ganapathisubramani 2019). Whereas, here we focus on inherently multi-scale buildings, more typical of dense high-rise urban landscapes. In other words, while formerly many carefully positioned roughness elements are needed to introduce the multi-scale characteristics of a real urban landscape, herein a single isolated building can be inherently multi-scale. To explore this topic, we employ fractal building models immersed in a deep boundary layer. The building models differ in their minimum length scales but have the same average frontal and plan area. Here we examine the drag force, pressure distribution, and wake characteristics induced by these buildings to inform whether changes to the flow structure are linked to the multi-scale nature of the buildings. To our knowledge, this is explored for the first time. Although differing from a surface formed from many interconnected and interlinked buildings (an urban rough terrain), the effect of a single isolated building on the boundary layer provides an understanding of the effects of added length scales to a single bluff body’s wake flow. In addition, we believe it is a gateway to exploring, by extrapolation, what their effects may be for a full rough wall.

In this manuscript, we discuss the experimental setup and facilities (Sect. 2) and results (Sect. 3) before drawing conclusions in Sect. 4.

## Experimental Facility and Details

### Experimental Facility and Boundary Layer Development

The experiments are carried out in the University of Surrey Environmental Flow Research Centre (EnFlo) ‘Aero’ wind tunnel. This tunnel’s working section covers 9000 mm (length) x 1060 mm (width) x 1270 mm (height). It is a closed-circuit wind tunnel with a maximum velocity of 40 $\mathrm {m\ s^{-1}}$ and a free-stream turbulence intensity below 0.1%. A Pitot-static probe at the tunnel inlet measures the free-stream velocity, $U_{ref}$. We consider a range of velocities, resulting in a Reynolds number range $Re_L=6.7\times 10^{3}-8.2\times 10^{4}$, based on the building height and the freestream velocity.

**Fig. 1:**
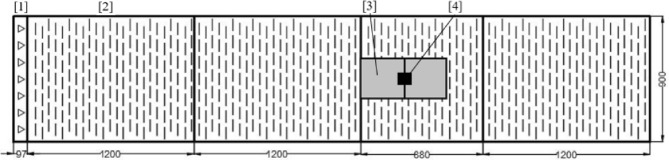
Wind tunnel showing location of the Irwin spires [1], roughness elements [2], shroud (see Fig. 4) [3] and building model [4]. Measurements in mm

Irwin spires, along with floor roughness elements are employed to produce a thick velocity profile similar to that of an urban boundary layer (Fig. 1). The spires have a height of 245 mm and a base width of 35 mm, with a centre spacing of 130 mm. The 2 mm tall and 8 mm wide floor roughness elements are spaced 16 mm apart in a staggered array. Using the approximation for the friction velocity of $u_{\tau } = \sqrt{-u'w'}$ (Castro 2007), where $u'$ and $w'$ are the fluctuating velocity components, this gives a boundary layer depth at the building location of $\delta \approx 221$ mm and $u_{\tau }=0.45$ $\mathrm { m\ s^{-1}}$, the latter being for the wake measurements which were all taken at 10 $\mathrm {m\ s^{-1}}$.

### Fractal Building Models

Two flat-surface buildings are used as reference cases (or base models). These very simplifed buildings, allow representation of multi-scale features in three dimensions. However, introducing multiple length scales - even if in only two dimensions - is a firm departure from the customarily employed cuboid shapes found in most prior urban flows literature. This can inform whether these additional length scales are needed to truly represent the urban landscape.

**Fig. 2:**
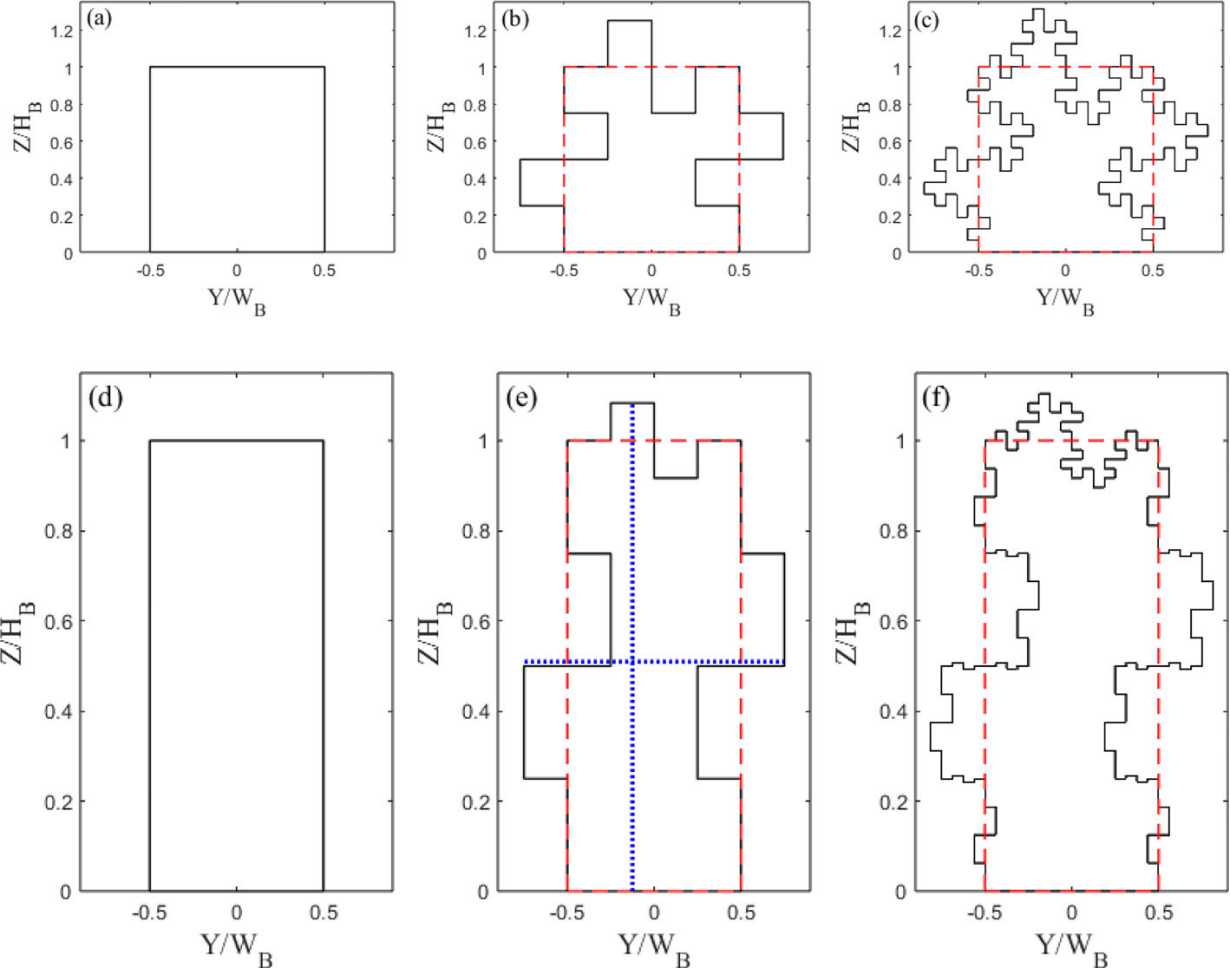
Building models normalised with mean height ($H_B$) and width ($W_B$) **a**–**c** Standard and **d**–**f** Tall, for (**a**,**d**) zero, (**b**,**e**) first, and (**c**,**f**) second iterations, with average width and height of models (red dashed lines). Blue dotted line in (**e**) shows an example of the maximum height and width dimension. Table 1 gives characteristics

Two simple cuboids case differ in their height-to-width ratios, *AR*. The Standard case has an of $AR=1$, whilst the tall square cylinder has an of $AR=3$ (hereafter Tall). From each form, two iterations of geometric complexity are added, each an order of magnitude more detailed or lower than the previous one. Thus, six building models are used (Fig. 2). All the Standard (Tall) models have a mean height ($H_{B}$) of 52 mm (90 mm) and width of 52 mm (30 mm). In an effort the keep the projected plan area constant, the depth, *D*, does change for each model with the increase in maximum width. This ensures that when the models are used in arrays, the plan area fraction remains constant which is an important morphometric parameter. The effects of the smaller length scales can be isolated by keeping these constant. As a result of keeping $H_B$ constant, all Standard and Tall models have an $H_{B}/\delta$ of 0.24 and 0.41, respectively. Modern tall buildings are increasingly tall (up to 400 m) (CTBUT 2024), and the urban boundary layer can be relatively shallow (of the same order of magnitude) in some conditions (stably stratified atmospheres) (Peng et al. 2017). The tall models, herein, take up a large portion of the boundary layer by design so their relative height ($H_B/\delta$) is chosen to reflect this. These building forms, though akin to natural geometries (i.e. trees) are inspired by several newly constructed and planned urban tall building designs, such as DJI Sky City, Atlantis The Royal Resort, Nexus, The David Rubenstein Forum and Antilia ( CTBUT (2024)).

**Fig. 3:**
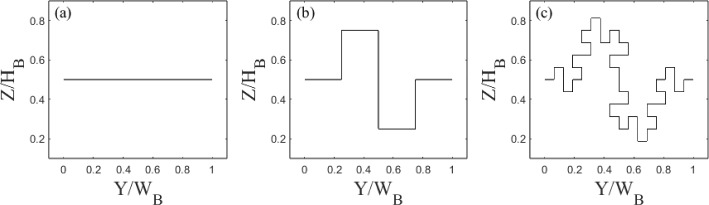
Generation of Minkowski sausage-type fractal geometry. Normalised with building average width ($W_B$) and average height ($H_B$)

Fractals are used in this work for convenience due to their self-similar properties. This allows one to increase the length scales of a shape while holding the area constant. The fractal iterations are obtained using a Minkowski Sausage-type generator (Mandelbrot 1983), as exemplified in Fig. 3, to decrease the minimum length scale of the models at each iteration. The results are the six building models in Fig. 2. Although more iterations are possible, the length scales rapidly become smaller than the sensor’s spatial measurement resolution rendering any differences undetectable.

The 3D printed building models used for drag estimation (Table 1) have embedded pressure ports (see Sect. 2.4) using grey resin from i.materlise (Technologielaan 15, 3001 Leaven, Belgium). It is important to contextualise the length scales characterising the fractal models used here with the flow scales. Of particular importance for the discussion in Sect. 3 are the smallest scales in the flow. These are assumed to be of the order of the Kolmogorov length scale ($\eta$), estimated using (Pope 2000):

1 $$\begin{aligned} \eta = \left( \frac{\nu ^3{\kappa }y}{u_\tau ^3}\right) ^{1/4}, \end{aligned}$$

where $\nu$ is the kinematic viscosity, $\kappa$ is the von Kármán constant, herein taken to be 0.41 (Castro 2007), and *y* is the wall normal distance. This gives a $\eta$, of 0.14$-$0.20 mm based on the friction velocity and range of wall-normal locations data are in this manuscript ($y/\delta = 0.5-1$). Hence the dissipative length scale in the flow is one order of magnitude smaller than the smallest length scales characterising the multi-scale models, which is of the order of 1 mm.

**Table 1:** Main geometrical statistics of the building models, where $H_{max}$ is the maximum height, $W_{max}$ is the maximum width, *D* is the depth and $L_{min}$ is the minimum length scale

Building model	$H_{max}$	$H_{B}$	$W_{max}$	$W_{B}$	*D*	$L_{min}$
Standard 0	52.00	52.00	52.00	52.00	36.00	36.00
Standard 1	65.00	52.00	78.00	52.00	24.00	13.00
Standard 2	68.25	52.00	84.50	52.00	22.15	3.25
Tall 0	90.00	90.00	30.00	30.00	36.00	30.00
Tall 1	97.50	90.00	45.00	30.00	24.00	7.50
Tall 2	99.38	90.00	48.75	30.00	22.15	1.88

All units in mm. See Fig. 2 for the full geometry

### Floating Element Force Balance

**Fig. 4:**
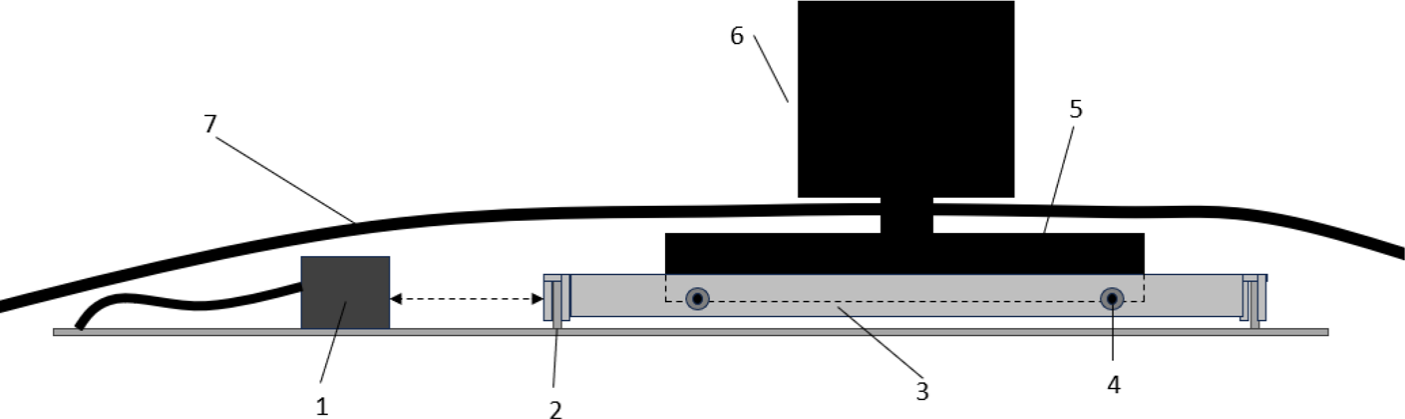
Sketch of FEFB showing [1] Optical non-contact displacement transducer, [2] spring allowing the system to move, [3] floating plate, [4] tightening screws, [5] 3D printed holder to fit models in, [6] 3D printed model with a cylindrical base to fit into the holder, and [7] the shroud (see Fig. 1). Sketch not to scale

To directly calculate the drag generated by the building models, a Floating Element Force Balance (FEFB) system is employed (Fig. 4). This has an optical non-contact displacement transducer (Micro-epsilon model optoNCDT 1420), accurate to $\pm 0.5 \mu$m, to record displacement of a floating plate, from which the drag force is calculated ( Hostick (2015)). The floating plate is held in position by a bespoke flexure system, designed to render the plate co-planar to the tunnel floor, eliminating any movement along the other degrees of freedom of the system (i.e. making it largely unaffected by moments by only subjecting it to linear forces in the direction of motion). Each data point is acquired over 18 s with a sampling frequency of 500 Hz. Measurements are also taken for 120 s to ensure the mean drift ($\pm 1\%$) and statistical convergence of the data ($\pm 1\%$) are achieved within the chosen sampling time. The mean drift is calculated by taking the mean of the signal over the first 18 s (the sampling time used for the measurements) and comparing it with the same measure over the last 18 s (out of the 120 s) to ensure no significant deviations were found. Statistical convergence was achieved by ensuring that the mean calculated over 18 s converged on the mean value calculated over 120 s. The force balance is pre- and post-calibrated to check for any drift, using a pulley system (hard-mounted to the tunnel floor) and a set of calibration weights up to 500 mN attached to the building model via fishing wire.

The measured drag, 10–200 mN, has a signal-to-noise ratio of 18.34 d**B** at the lowest tested speeds and a minimum of 48.96 d**B** for all Reynolds number independent results. This is orders of magnitude lower than the statistical error of the FEFB oscillating in the turbulent flow and thus not considered to be of statistical significance. The calibration linearity is satisfactory ($R^2\approx 1$).

The FEFB sits on the tunnel floor, covered by a low-profile shroud to minimise its effect on the flow (Figs. 1 and 4). This results in the buildings being raised by 20 mm above the tunnel floor, leading to a drag overestimation from the accelerated flow. From the velocity profile measured at the location of the FEFB, we estimate a 22% difference in momentum of the flow is present due to the raised wall, which needs to be accounted for. This is calculated by integrating the vertical velocity profile from both the tunnel floor and the FEFB, to compare the difference in momentum in the flow across the two measurement ranges. The drag from the FEFB is then scaled down by this difference to allow for a fair comparison between the two methods.

### Static Pressure Ports

An independent measure of the drag exerted by the buildings is from the pressure measurements (Cheng and Castro 2002). The 3D printed models have static pressure ports with an internal barb connected to a length of silicone tubing, fed out of the building base and the wind tunnel floor to multiple 48-port connectors. The connectors are fed with more tubing to two 64-channel pressure scanners (SSL model DPS14–160P, Guildford, Surrey) with a full-scale range of 160 Pa and an accuracy of 0.25% full scale. Each data point has a sampling rate of 100Hz for 60 s. The ports are located on the building model’s front and rear faces, but their numbers depend on space availability on the model base: Standard 0 iteration model has 60 static pressure ports, Standard 1 has 64, and Standard 2 has 80. As the Tall models have greater base restrictions, iteration 0 and 1 models have only 42 and 52 ports, and Tall 2 has 66 ports.

The pressure ports distribution for the base models is designed to be inhomogeneous to capture the increased pressure gradients typically seen at the top of the buildings (Castro and Robins 1977). Given it was less clear what the pressure distribution would look like for the iteration 1 models, the ports were designed to be more equally spaced. For Tall 2 models, given the geometric constraints it is not physically possible to house pressure ports in the very top fractal spaces, so to attempt to capture the pressure force as accurately as possible more ports are placed in proximity of this area. For Standard 2, all outside fractal spaces could contain a port due to the increased size of the smallest length scale. This resulted in an ad-hoc distribution of pressure ports for each model. While *a priori* it is unclear whether the spatial resolution of these ports is sufficient, we explore and discuss this topic (Sect. 3.1.2).

The pressure on the front and rear face of each building model is integrated to give a total force acting on the building ( Cheng and Castro (2002)). Subtracting the force on the rear face from the front gives the approximate drag on the model, allowing comparison with the FEFB measurements and an indication of necessary spatial resolution (i.e. number of pressure ports) to accurately capture the drag of multi-scale tall buildings. A linear interpolation is used between the pressure ports across a grid which matched the geometry of the models. Different grid resolutions are tested to ensure the results are grid-independent. The pressure port readings are only interpolated across ports in the same area of the building e.g. port at the bottom left of Tall 1 model (co-ordinate (0.14, 0.075) in Fig. 8) would not influence the port at its top left (co-ordinate (0.2, 0.79) in Fig. 8). The interpolation helps to provide a more realistic visualization of the pressure but has very little effect (one order of magnitude lower than the quoted uncertainty of the pressure measurements) on the overall drag result. This is to say that the maximum percentage difference in the overall pressure due to the different integration methods (linear, nearest, natural) is far lower than that uncertainty from the pressure scanner itself. As for the uncertainty due to the pressure ports’ location, this remains unassessed (directly at least) as it is not possible to place ports in certain geometric locations (e.g. at the top of Tall 2 model) and compare the results. However, the comparison of the pressure results with the FEFB in Sect. 3.1.2 should offer an acceptable indirect estimation of the magnitude of this uncertainty.

### Wake Measurements

**Fig. 5:**
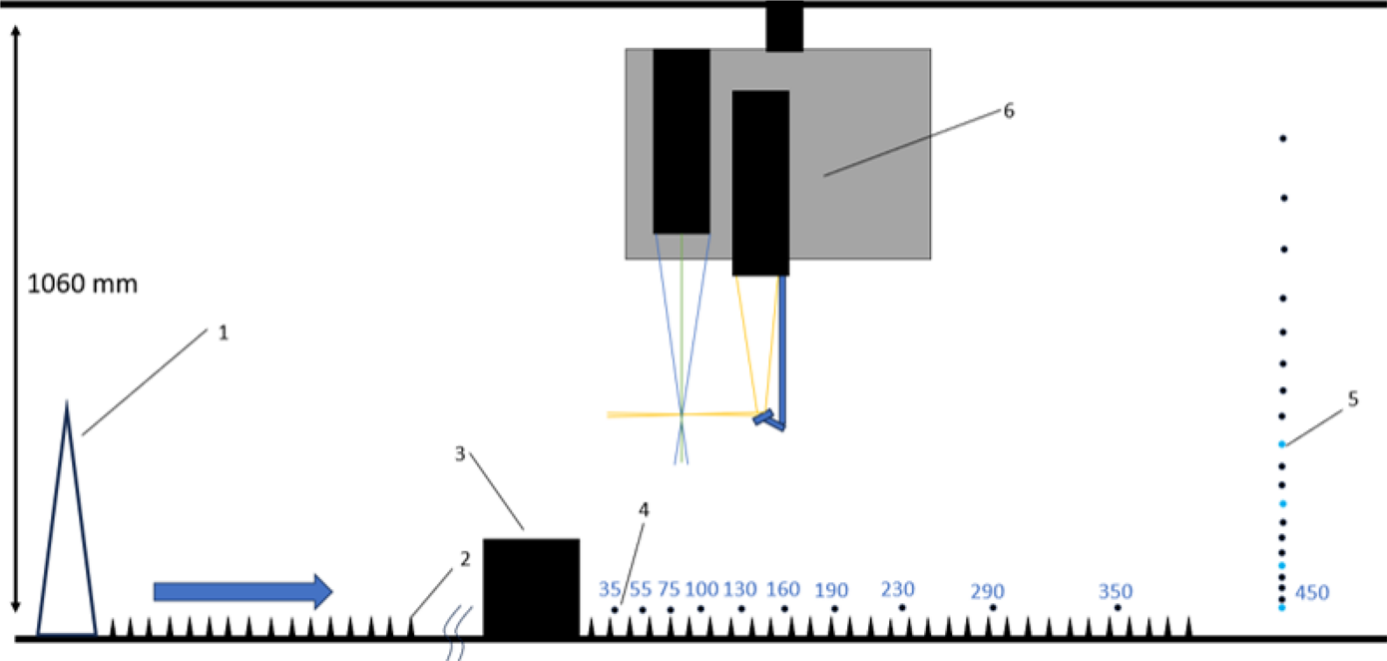
Tunnel side view ( showing inlet dimensions. LDA set up with [1] Irwin spires, [2] floor roughness, [3] building model, [4] streamwise location of vertical and lateral profiles, [5] example of vertical measurement profile (black dots) with height of lateral profiles taken (blue dots) ($$Z = $$ 22.5 mm, 45 mm, 82.5 mm and 120 mm for the Tall models and $$Z = $$ = 13 mm, 26 mm, 39 mm and 68 mm for the Standard models, [6] LDA probe shroud, [7] mirror, [8] LDA probes. All measurements in mm. Not to scale

A three-component Laser Doppler Anemometer (LDA, Fibreflow, Dantec Dynamics, Denmark) is used to simultaneously measure the mean (*U*, *V*, *W*) and fluctuating $(u', v', w')$ velocity components, corresponding to the streamwise (*x*), spanwise (*y*) and wall-normal (*z*) directions, respectively. The building models are placed 2000 mm downwind of the test section inlet (Fig. 5). A cartesian coordinate system (*X*, *Y* and *Z*) is employed for the wake measurement with origin at the centre of the building in *X* and *Y* directions, and ground level in *Z* direction.

The laser beams are converged by a 300 mm focal length lens. An acquisition frequency of 500 Hz is set as the minimum target for the flow seeder. Each data point is recorded for 30 s to allow for sufficient statistical convergence while constraining experiment run time. For each building model, vertical profiles are taken at several streamwise distances 37 mm $$\le X \le 450$$ mm, for $$Z= 13 $$ mm to 250 mm. At each *X* location, lateral profiles are taken at four heights ($$Y=-150$$ mm to 150 mm) that depend on the model (Fig. 5. The *U*, *V*, and *W* component lasers are set to a power of 200 mW, 350 mW and 150 mW, respectively. The Measurement resolution of the LDA is 0.14 mm.

## Results and Discussion

### Drag Measurements

#### Floating Element Force Balance

**Fig. 6:**
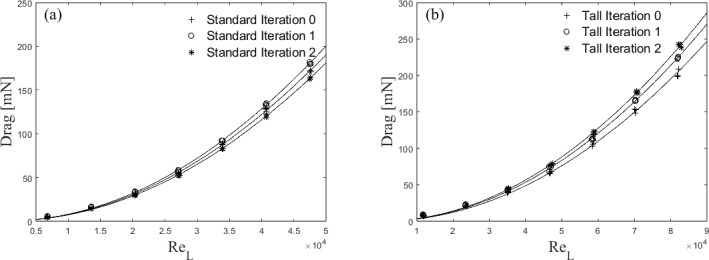
Drag [mN] as a function of $Re_{L}$ for **a** Standard and **b** Tall building models showing measurements (symbols) with best-fit lines. Calculated as discussed in Sect. 2.3. Note there are 3 data points for each building model at each Reynolds number

The directly calculated drag on each building (Sect. 2.3), show good repeatability (Fig. 6), with three different runs concurring within 4%. A second-order polynomial fit using least square (Fig. 6) is employed. Each fractal iteration (Fig. 2) shows a clear change in drag, with the Tall building models experiencing a larger drag than the Standard models (up to 20%). For the Tall buildings, each iteration has a non-negligible increase in drag, consistent with Nedić et al. (2013). Surprisingly, the Standard model’s second iteration (Standard 2) shows a drop in drag below that of the base cuboid model, despite an increase in the first iteration (Standard 1).

**Fig. 7:**
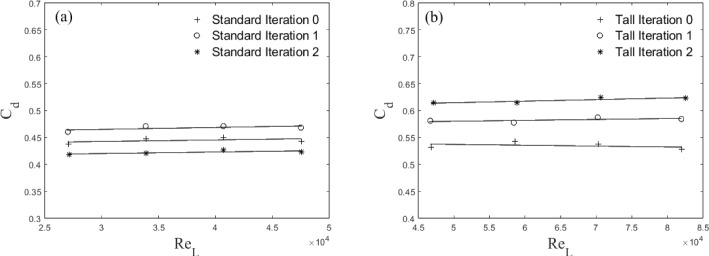
Drag coefficient as a function of $Re_{L}$ for **a** Standard and **b** Tall building models with $C_{d}$ decreased by 22% (Sect. 2.3). Note both X and Y scales differ between (**a**) and (**b**)

In non-dimensional terms, the variation of drag coefficient ($C_d$) with $Re_L$ is shown in Fig. 7. $C_d$ is calculated based on the frontal area of the models, *A*, the air density, $\rho$, and the freestream velocity (see Eq 2):

2 $$\begin{aligned} {C_d = \frac{D}{0.5\rho U_{ref}^2A}} . \end{aligned}$$

This is presented to demonstrate that, for each building, the drag coefficient is independent of the Reynolds number in the range measured for Standard models ($2.5\times 10^4< Re_L < 5\times 10^4$) and Tall models ($4.5\times 10^4< Re_L < 8.2\times 10^4$), respectively. These figures also further help to quantify the effect of the addition of smaller length scales. The inherent oscillation of the FEFB around the mean drag value during operation can result in misleading large error bars (hence omitted). However, despite this large variation, the difference in the mean drag across each iteration was confirmed to be significant (95% confidence interval) with a t-test (Herzog et al. 2019).

For the Tall building models $C_d$ increases by approximately 8% for Tall 1 and 13% for Tall 2, relative to the Tall 0 case. Nedić et al. (2013) using plates in shear-free flow has a similar trend, with an increase in drag coefficient for their first couple of fractal iterations of $\approx 7\%$. The Standard building shows a different trend in $C_d$ with the addition of length scales. While Standard 1 has an increase in $C_d$ from Standard 0 of 5%, there is a decrease in $C_d$ for the Standard 2 case by $\approx 5\%$. Although this decrease in drag for Standard 2 is somewhat unexpected, it is consistent with Nedić et al. (2013) who used the same Minkowski Sausage-type fractal generator, at comparable minimum length scale and Reynolds number. They ascribed this to a change in the wake volume, with a reduction in wake size causing a reduction in drag. A similar process is expected to be at play here, and it is consistent with wake profiles observed from these models (not shown). The question that naturally arises is, what process causes the wake volume reduction? One hypothesis is that the smaller lengthscales characterising model S2 partially redistribute the energy toward the dissipative range (or higher frequencies), feeding the Richardson-Kolmogorov cascade process and facilitating the wake recovery. This is not dissimilar to the findings for fractal-generated turbulence with multiscale passive grids, which typically produce faster turbulence decays following an enhanced kinetic energy immediately downstream of the grids (Hurst and Vassilicos 2007, Valente and Vassilicos 2011, Thormann and Meneveau 2014). We present some evidence of this process in Fig. 21, however, this alone would not explain the different trends in the drag reported for S1 and S2. Two other points are worth noting here. The first relates to the maximum height of the models adopted that changes significantly from S0 to S1 (52 mm to 65 mm) but less so between S1 and S2 (65 mm to $\approx 68$ mm). In this respect, a comparison between S1 and S2 is much fairer if one is interested in purely isolating the effect of the added lengthscales, as an increase in the maximum building height would expose it to higher momentum fluid, likely increasing drag (see a further discussion in the following). The second point is around the shape change across models S0 and S1/S2. Introducing the first fractal iteration (from S0 to S1) significantly changes the overall shape of the building, but the second iteration (from S1 to S2) does not. In the former, it is likely that streamwise vorticity is replaced partly by its spanwise wall-normal components, which would be characterised by a large-scale organisation (based on the large geometric different scales introduced in the model S1). The iteration from S1 to S2 is instead much more intimately linked with the small scales due to the more subtle geometric changes, which were shown to disrupt coherent structures and affect drag in (Gehlert P 2020). It is impossible, within the limits of the current dataset, to firmly identify any of the above reasons as the main corporate responsible for the trend in drag for the standard models and further investigation would be needed for these wake flows, as identified in Vassilicos (2015), before a firm conclusion can be reached. It is likely that a non-trivial combination of all the above is at play here. As the tall buildings do not show this peculiar non-monotonic change in drag as a function of fractal iteration, we assume this wake phenomenon is not occurring. This may be because for the Tall case it is not possible to have the full fractal pattern around the whole model due to manufacturing constraints, so the smallest length scales only occur near the top of the buildings (see Fig. 2). See further discussion in Sects. 2.4 and 3.2.

It must also be noted that by introducing smaller length scales employing the Minkowski Sausage-type fractal generator, we are inherently slightly changing the maximum height of the building across iterations (see Table 1). This change must affect the generated drag. We estimate the increase resulting from purely exposing the top of the higher-iteration buildings to higher flow speed (given the increase in maximum height of the models – analogous to Sect. 2.3 discussion), but this is insufficient to explain the changes in drag recorded across cases, as it only accounts for a small fraction of the drag increase (less than 0.5% of the overall drag increase from Tall 0 to Tall 1). This change would be even smaller for the second iteration as the maximum height changes by a smaller percentage. Furthermore, this effect alone would not account for the trend in drag change encountered by the Standard models. Hence, we comfortably conclude that the introduction of the length scales is, indeed, responsible for the significant effect seen on the aerodynamic properties.

#### Pressure Results

**Fig. 8:**
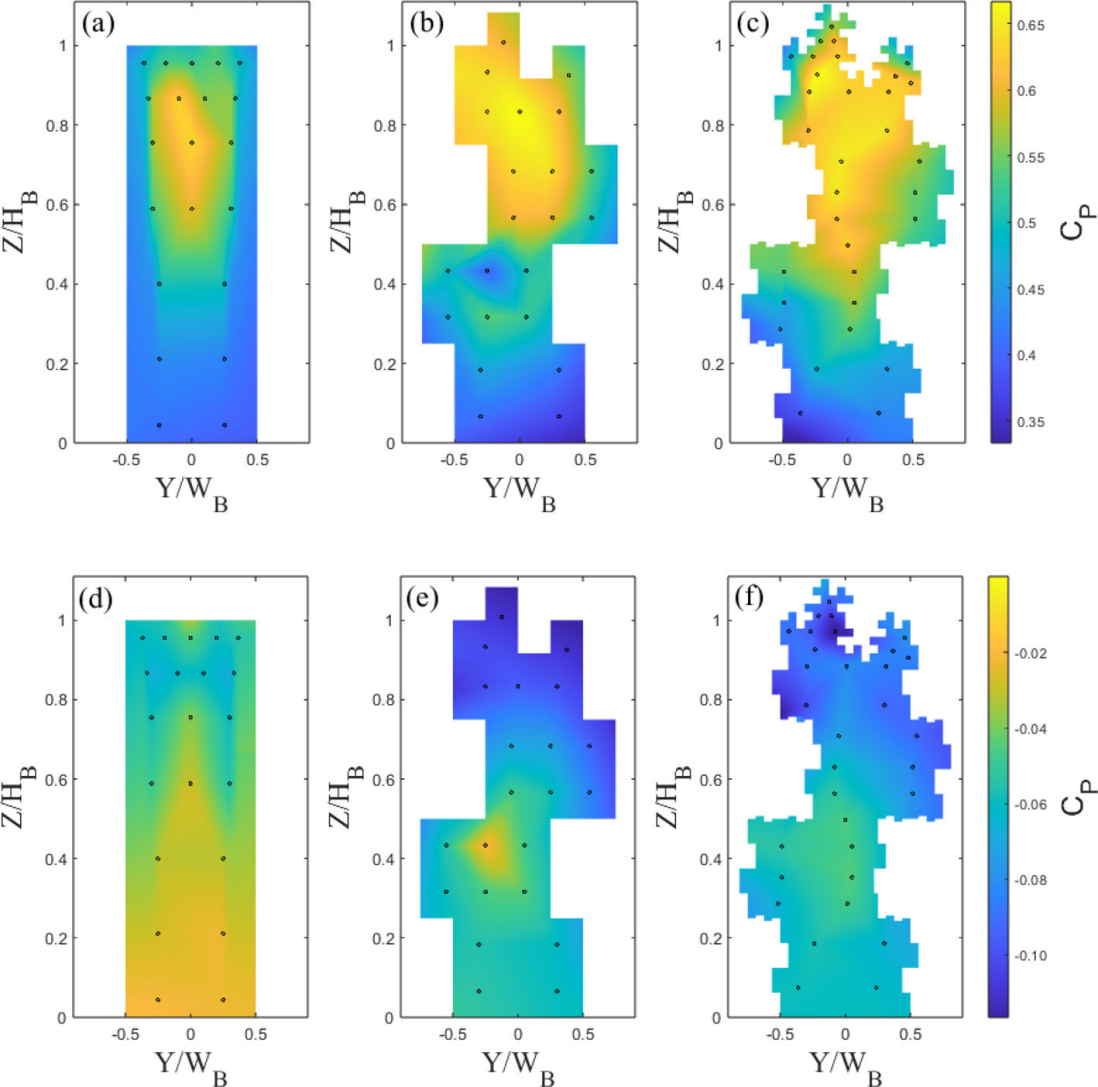
Static pressure ports locations (dots) and pressure coefficient, $C_P$, contours for Tall building models showing **a**–**c** front face and **d**–**f** rear face for iteration (**a**,**d**) 0, (**b**,**e**) 1, and (**c**,**f**) 2. $Re_L = 8.2 \times 10^4$

**Fig. 9:**
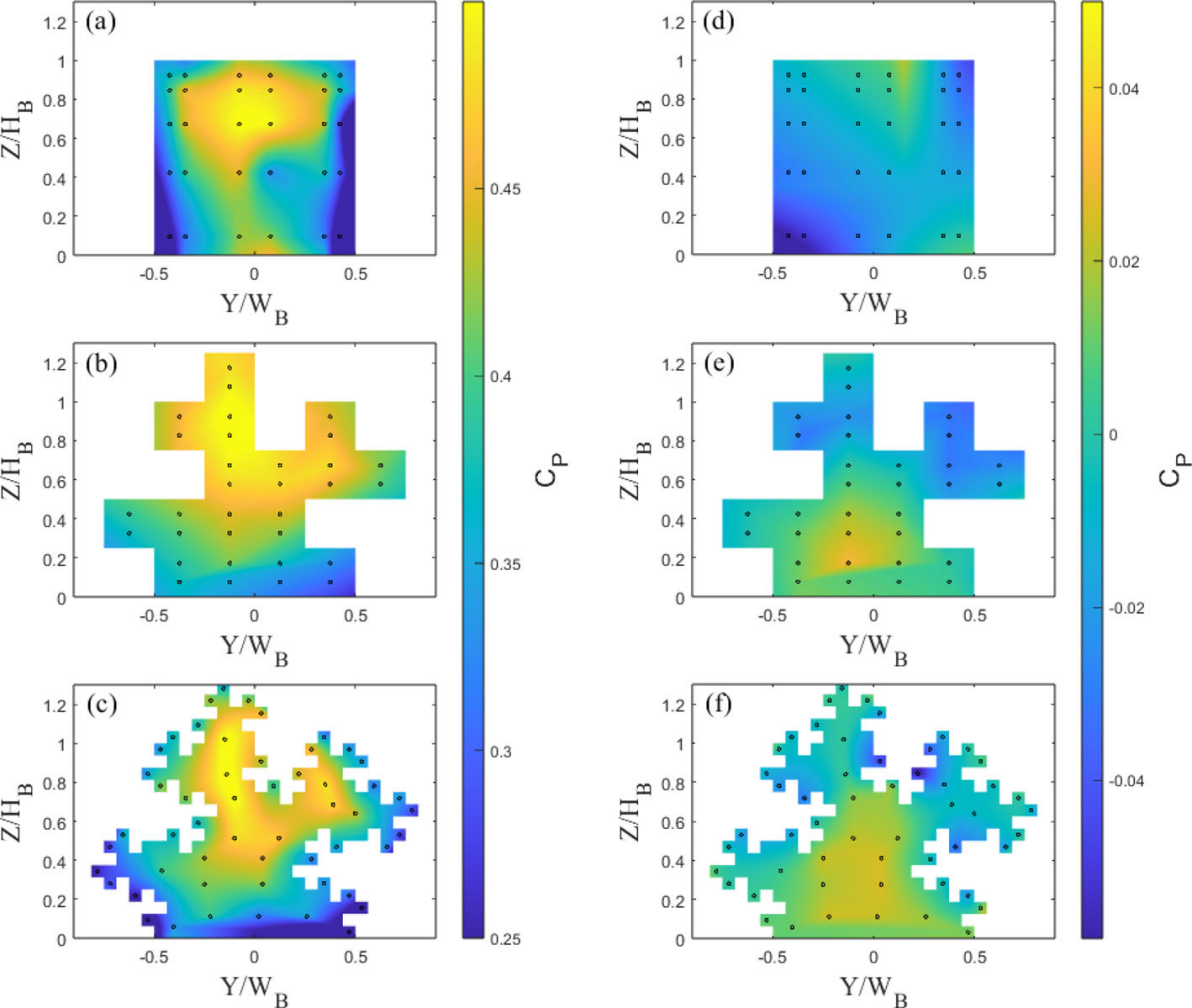
Static pressure ports (dots) and pressure coefficient, $C_P$, contours for Standard building models showing **a**–**c** front face and **d**–**f** rear facet for iteration (**a**,**d**) 0, (**b**,**e**) 1, and (**c**,**f**) 2. $Re_L = 5 \times 10^4$

To gain insights into the force distribution on the front and rear faces of the buildings, the pressure contour across each building (interpolated for visualisation purposes) is shown in Figs. 8 and 9. The drag force is determined using (Cheng and Castro 2002):

3 $$\begin{aligned} {D = \int _{A}(p_f - p_b)dA}, \end{aligned}$$

where $p_f$ and $p_b$ are the pressures on the front and rear faces of the model respectively The Tall and Standard front face of the building’s maximum pressure (hence drag force), between $0.7H_B< Z < 0.8H_B$, is in line with expectations for the base models ( Castro and Robins (1977)). The variation of pressure with height on the front and rear faces (for Standard 0 and Tall 0) is also consistent with previous literature (Castro and Robins 1977; Surry and Djakovich 1995; Lim et al. 2009; Kozmar 2021). The drop in pressure on the rear faces with each iteration, particularly for the Tall models (Fig. 8), leads to an increase in drag. However, Standard 2 shows a decrease in front-face pressure from Standard 1. It appears the small outer protuberances produce more regions of lower pressure, contributing to the overall decrease in drag.

Using the Sect. 2.4 drag coefficient procedure, we can compare the values for each building model with those from the FEFB (Sect. 4). Overall for all buildings, the pressure data estimates have a drag within 7% of the FEFB (Table 2). Importantly, the drag on the Tall buildings follows a very similar trend to the FEFB results, with an increase of 11% and 15% for Tall 1 and 2 (cf. Tall 0), respectively. This suggests the number and location of the pressure ports (and their spatial resolution) are enough to capture the change in drag between iteration 0 to 1, but perhaps insufficient to capture the full change from iteration 1 to 2. The Standard models also have a similar trend to the FEFB results, with an increase of 20% from Standard 0 to 1, and a decrease to Standard 2 of 2% (cf. Standard 0). Again, this shows the pressure resolution is enough to capture clear changes in drag between each iteration, but potentially not to fully capture the change around the Standard 2 case. These discrepancies are clearly a consequence of the limited pressure resolution because of the internal space limitations of the models. Nevertheless, these results are encouraging as they suggest that drag (i.e. velocity scaling) can be inferred in much larger building arrays using only the static pressure ports, when a FEFB is not available or would prohibitively increase the experimental data collection.

**Table 2:** $C_d$ comparison between FEFB and pressure measurements

Model ID	$C_{d_{FEFB}}$	$C_{d_{p}}$	$\Delta \%$
Tall 0	0.54	0.55	2
Tall 1	0.58	0.61	5
Tall 2	0.62	0.64	3
Standard 0	0.44	0.41	7
Standard 1	0.47	0.50	6
Standard 2	0.42	0.40	5

### Wake Measurements

#### Mean Velocity Fields

**Fig. 10:**
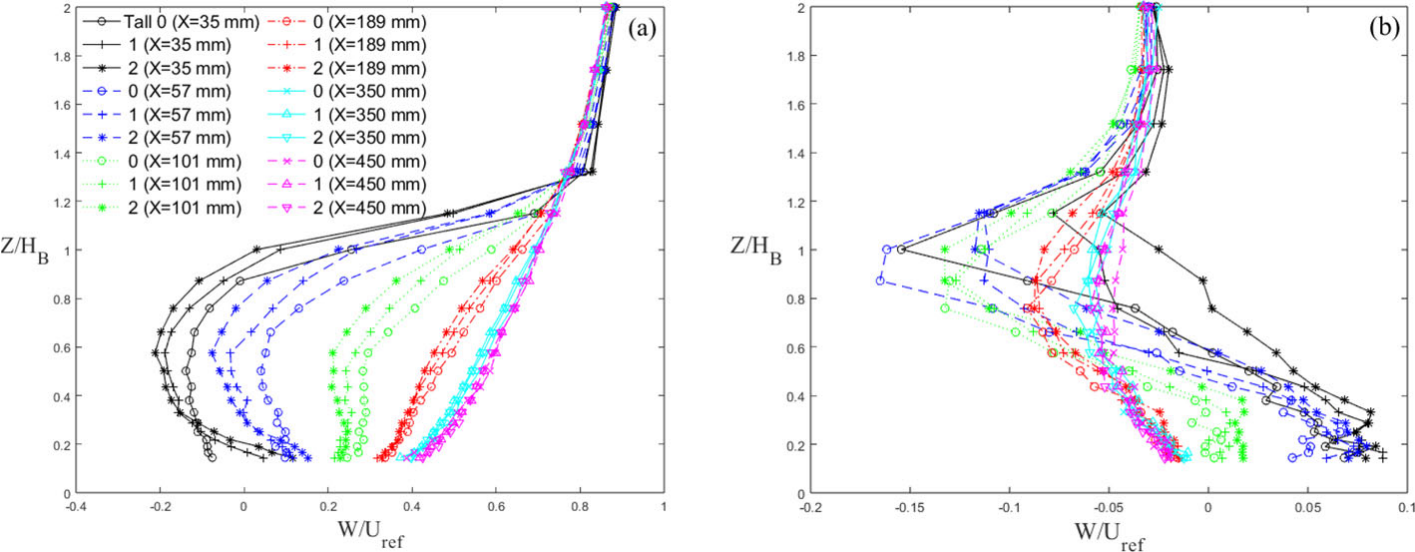
Vertical streamwise (**a**) and vertical (**b**) velocity profile of wake behind three Tall building models (symbol) normalized by mean building height with distance (line style)

**Fig. 11:**
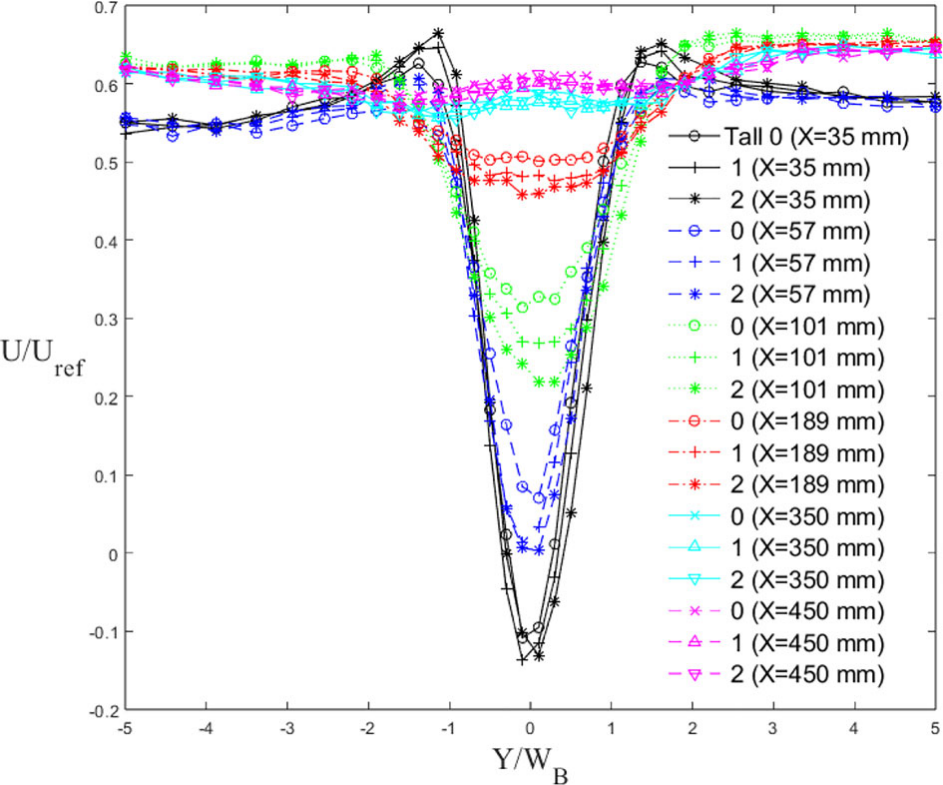
Lateral velocity profile of wake behind Tall building models normalized against average building width for the three models (symbol shape) with distance (line style). Data are taken at the models’ mid-height

The vertical (Fig. 10a) and lateral (Fig. 11) velocity profiles of mean streamwise (*U*) and vertical (*W*) velocity (Fig. 10b) for the Tall building models are non-dimensionalised with the inlet freestream velocity ($$U_{ref}$$). $$U_{ref}$$ is measured by a Pitot tube at the inlet of the tunnel far above the spires to ensure it is in the freestream. The vertical profiles are at the building centreline ($$Y=0$$), whereas the lateral profiles are taken at mid-building height ($$Z=0.5H_B$$). From the vertical velocity profiles, it is observed the wake is fully recovered by $$X=450$$ mm (Fig. 10a). Strong regions of re-circulation are also observed at $$X=33$$ mm and 55 mm peaking at $$Z/H_B=0.5-0.6$$, with the higher iteration buildings producing a more pronounced re-circulation region. The vertical streamwise velocity profiles in the near-wake region show the sensitivity of the flow to the local length scale of the buildings. For example, at $$X= 35 $$mm, the streamwise velocity measured nearest to the ground is negative for Tall 0 (base model), while it is positive for Tall 1 and Tall 2. This shows that the length of the recirculation region is governed by the building width at the local considered height. This is confirmed at $$Z=0.5H_{B}$$, where the strength of the wake is strongest for Tall 2, as this is the height with the largest width (cf. Tall 0 and Tall 1, Fig. 10). The vertical velocity in Fig. 10b follows the expected trend with a maximum and minimum at the top and base of the building respectively, due to the recirculation region developing behind the models. The effect of the fractal iteration is visible in the near wake (blue and black profiles) but quickly becomes less obvious furhter downstream. Regions of velocity ‘overshoot’ just above the mean building height are seen at $$X=35$$ mm (Fig. 11), indicating flow acceleration over the buildings, due to local blockage. These features are also present for Standard building models (not shown), with clear differences seen across cases and fractal iterations. This is important as between the respective base models and the first iteration, the buildings undergo a clear geometrical shape change, while the second iteration models are very similar in shape to the first iteration models, yet still result in noticeable changes in the wake flow. This implies that the differences across cases are not purely due to a change in building shape, but rather to the addition of the length scales. This is true for virtually all results discussed in this work. The lateral velocity profiles at $$Z=0.5H_B$$ do not show any significant difference between each iteration (Fig. 11). However, the velocity deficit at a given streamwise location is observed to increase with an increase in iteration, in line with the drag results presented in Sect. 3.1.

#### Wake Spreading

**Fig. 12:**
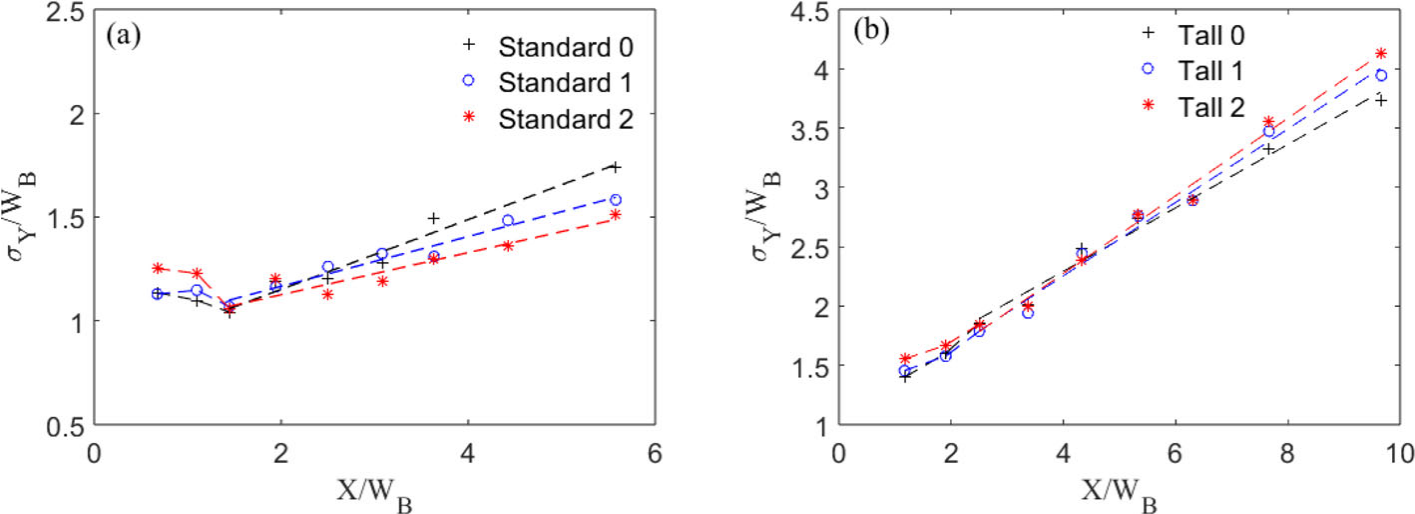
Lateral wake spread normalised by the mean building width for the three **a** Standard and **b** Tall models. Data at models’ mid-height

**Fig. 13:**
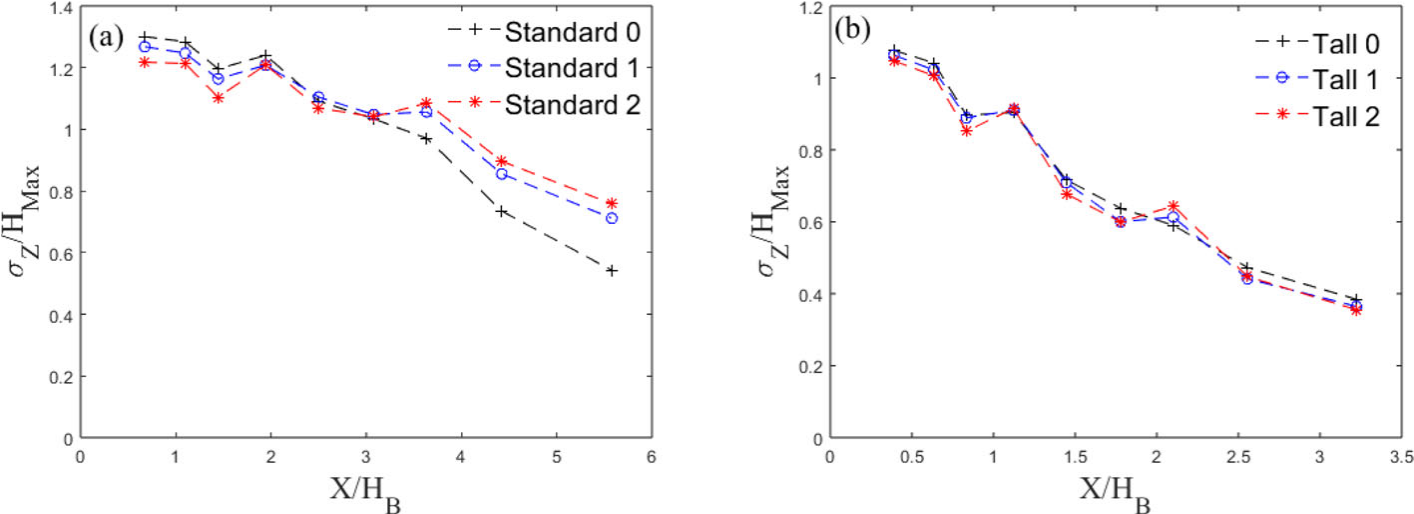
Vertical wake spread normalised by maximum building height for the three **a** Standard and **b** Tall models

To understand the effect of the fractal geometry on the growth of the wake behind the building, we analyse the wake width in the lateral ($\sigma _\textrm{Y}$) (Fig. 12) and vertical directions ($\sigma _\textrm{Z}$) (Fig. 13). We define the lateral spreading of the wake as the distance between points on either side of the centre line when the velocity deficit has recovered to 50% of the maximum deficit ( Liu et al. (2002); Khan et al. (2018); Mishra et al. (2023)), with the maximum deficit being the difference between the local minimum ($U_{min}$) and local undisturbed velocity ($U_0$).

For the Standard buildings, the wake does not initially spread (Fig. 11) in the region $X\le 2W_B$. This is due to the strong flow recirculation seen downstream of the models. In accordance with literature (Liu et al. 2002; Khan et al. 2018; Mishra et al. 2023), further downstream the wakes show a nearly linear growth in *X*. For wake numerical modelling purposes, it is interesting to note that, for all cases considered here, the wake growth deviates notably from the behaviour assumed in classical atmospheric dispersion modelling (Robins and McHugh 2001), which predicts the wake to follow the power law growth as $\sigma _\textrm{Y} \sim x^{1/2}$. It can be argued this deviation is ascribable to different approach flow conditions. A linear wake growth ($\sigma _\textrm{Y} \sim x^{1}$) has been found when ambient turbulence levels (RMS of the streamwise velocity fluctuation, $u_{rms}$) are comparable to the strength of centreline velocity deficit (Eames et al. 2011). This is consistent with the findings in this work (compare Fig. 11 with 15). Similarly, wake linear spreading is reported downstream of isolated tall buildings immersed in a deep boundary layer (Mishra et al. 2023).

The wakes for the Tall models spread faster ($2 \le \sigma _\textrm{Y}/W_B \le 4$) compared to those of the Standard models ($1 \le \sigma _\textrm{Y}/W_B \le 1.5$). The rate of lateral wake spreading, $\frac{d\sigma _{y}}{dX}$ for Tall 0 (0.33) is consistent with that of Mishra et al. (2023) (0.32) giving confidence in the base model results. For Standard 0, equally the initial wake contraction and then its linear growth is comparable to that of Khan et al. (2017) at high Reynolds numbers. The second iteration models have a wider near wake for both the Tall and Standard models. The initial difference in wake size is expected as the buildings have different maximum dimensions, but by the third measurement point ($X/W_B= 1.5$), i.e. beginning of linear spreading) the wakes have virtually the same width. Downstream, Standard 0 has the greatest spread of the Standard models, whereas it is Tall 2 for the Tall models.

The vertical wake spread is more complex to evaluate due to the strong regions of flow recirculation behind the models, which prevents the velocity at the lowest point from being conveniently used as a reference value. Instead, we use a reference velocity value of the freestream velocity, at which points all velocity profiles had collapsed. The vertical height of the wake is then calculated as the height at which the velocity reaches 50% of the reference value (Fig. 13). While this is not a direct calculation of the actual wake height, it gives a good indication of the vertical spreading.

**Fig. 14:**
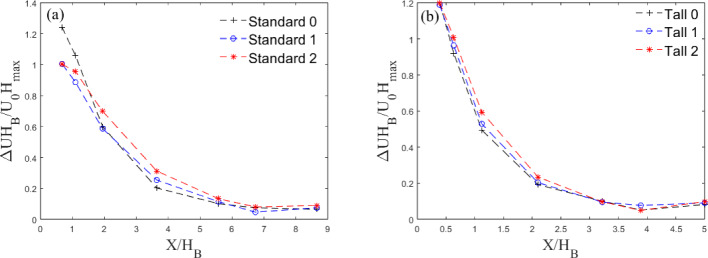
Wake deficit normalised by maximum building height with distance normalised by mean building height for the three **a** Standard and **b** Tall models at $Z/H_{B}=0.5$

Both the Standard and Tall models show a wake spread behaviour that scales with the maximum height of the building models, suggesting this is an appropriate scaling factor. From Fig. 10, is it clear that the vertical wake is capped by the strong shear layer developing at the building roof level, this results in a wake contracting (Fig. 13) as it gets pushed downward by the growing shear layer depth. This is in accordance with Mishra et al. (2023). Standard 0 spreads more initially (Fig. 13a), likely due to the weaker shear layer at the roof level. This gives a weaker collapse in the vertical wake spread initially when compared with Standard 1 and 2 up to $X/H_B = 2$. After $X/H_B = 4$, the standard models show more spread in the data. This is expected to be a result of the larger differences in rate of decay seen between each iteration for the standard models (see Sect. 3.2.3 and Fig. 14a). Overall all models show a similar decay in vertical spreading when scaled with the maximum building height.

#### Wake Deficit and Recovery

The longitudinal wake deficit (Fig. 14) is calculated similarly to its lateral counterpart (Sect. 3.2.2). Here, scaling uses the maximum and the mean height of the buildings and the local velocity, to collapse the curves well, particularly for the Tall models (Fig. 14a).

The deficit follows the power law decay. To analyze the rate of deficit recovery for each iteration, power law decay curves are fit to the data in the form $y=ax^{b}$ where *b* indicates the recovery rate. *b* is observed to be $-$0.98, $-$0.93 and $-$0.90 for Tall 0, Tall 1, and Tall 2, respectively, and $-$0.92, $-$0.82 and $-$0.72, respectively, for Standard 0, Standard 1, and Standard 2. The quickest wake recovery is for the base iteration of both the Tall and Standard models. This is linked to the enhanced turbulent mixing and flow entrainment within the wake generated by the higher turbulent fluctuations in the wake of iteration 0 models (further discussed in Sect. 3.2.4).

As for wake growth (Sect. 3.2.2), it is of interest to compare the wake recovery rate to two-dimensional wake theory (Counihan et al. 1974), as this forms a base for the atmospheric dispersion modelling system (ADMS)(Robins and McHugh 2001). While the ADMS model predicts the recovery rate to be $b=-0.5$, we observe this to be closer to $-1$ for both Standard and Tall models. Similar behaviour is observed in flow around a wall-mounted cube in a fully developed channel flow (Yakhot et al. 2006), as well as for wind turbine wakes in uniform flow (Johnson et al. 2014). This faster decay of the wake is attributed to the presence of ambient turbulence, with the centreline wake deficit found to follow $x^{-1}$ (Eames et al. (2011)). Notably, $b\rightarrow -1$ as the aspect ratio of the model increases (compare Standard 0 to Tall 0), rendering their wake more three-dimensional and causing it to deviate from two-dimensional wake theory. Perhaps unsurprisingly, these results suggest modifying the ADMS model would help more accurately capture three-dimensional effects.

The wake recovery rate is also observed to decrease with the introduction of smaller length scales (see variation in *b* from Standard 0 to Standard 2 and Tall 0 to Tall 2. This is somewhat surprising as introducing smaller scales could facilitate the energy cascade towards the dissipative range resulting in quicker recovering wakes ( Pope (2000), p.182–190) – though this is also a function of the magnitude of the turbulent fluctuations, as discussed further in Sect. 3.2.4.

Overall, tall building wakes show a quicker recovery. This is probably linked to the higher bulk velocity at $Z=0.5H_B$, given $H_B$ is shorter for Standard buildings (Table 1). The freestream flow penetrates quicker in the wake of the flow resulting in higher lateral wake width and faster recovery of the wake.

#### Fluctuating Velocity Fields

**Fig. 15:**
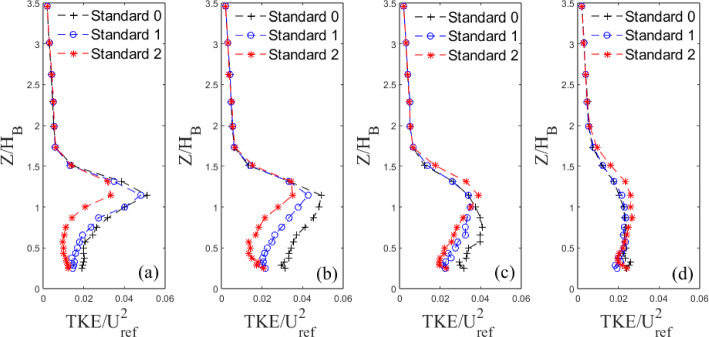
Vertical non-dimensional TKE profiles behind Standard building models at four example downstream locations ($X/H_{B}$=) **a** 0.67, **b** 1.10, **c** 1.94, **d** 3.63

**Fig. 16:**
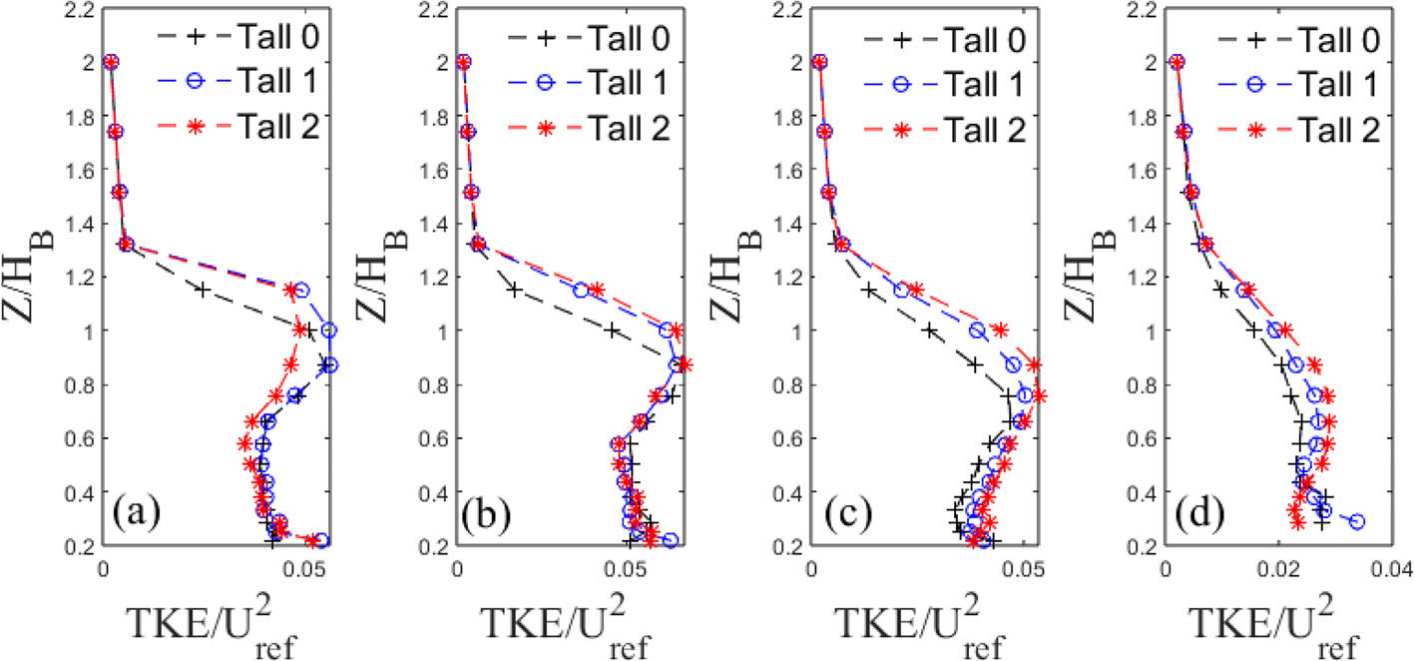
Vertical non-dimensional TKE profiles behind Tall building models at four example downstream locations ($X/H_{B}$=) **a** 0.39, **b** 0.64, **c** 1.12, **d** 2.05

To further investigate the effects of model length scales on the wake properties and offer an interpretation of the Sects. 3.2.2 and 3.2.3 results, we next consider the turbulent fluctuating fields. Variations in turbulent kinetic energy, TKE=$\frac{1}{2}(\overline{u'^2}+\overline{v'^2}+\overline{w'^2})$, with height are analysed for both building models at different *X* locations (Figs. 15 and 16).

For the Standard 0 case, the TKE at $0.67\le X/H_B\le 1.94$ is higher than for Standard 1 and Standard 2 (Fig. 15) up to the maximum height of the models. This is surely due to the different near-wake turbulent characteristics and coherence, which are highly dictated by the model geometry and its change across the wall-normal direction). Changes across cases are confined below the model roof level, with all cases showing concurrence above this height. After $X=2H_{B}$, the turbulence strength of the Standard base iteration model drops below that of the first iteration which, in turn, drops below second iteration, though marginally. The Tall models show a negligible change in TKE for $X/H_B\le 1$ between each iteration (Fig. 16 a, b). After this point, the TKE follows the same trend as the Standard models, with Tall 0 levels being below Tall 1, which in turn is below Tall 2.

Overall, the TKE is greater in the wake of the base models in the near wake, but as expected, it dissipates at a faster rate due to the higher turbulent mixing and flow entertainment; this confirms the trends discussed in the previous sections. Nedić et al. (2013) found similar results using fractal plates as fractal dimensions increased (adding smaller scales) the turbulence fluctuations behind the plates decreased and recovered slowly further downstream. However, as they only examined streamwise fluctuations, one could argue whether energy could have simply been redistributed in the other directions. Here, as we measure the three-dimensional TKE this energy redistribution process can be safely excluded.

**Fig. 17:**
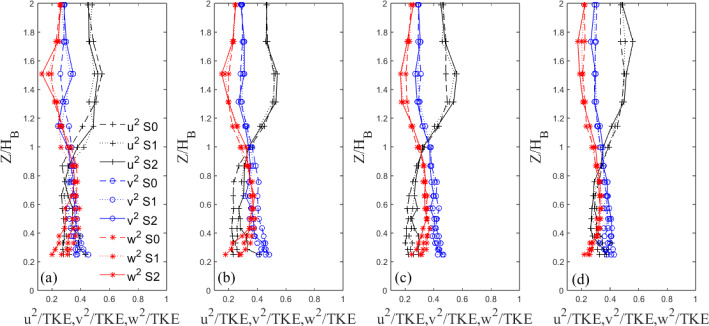
Vertical profiles of velocity fluctuations a four example downstream locations ($X/H_{B}$=) **a** 0.67, **b** 1.10, **c** 1.94, **d** 3.63. Here ’S’ is used for Standard to make the figure more readable

**Fig. 18:**
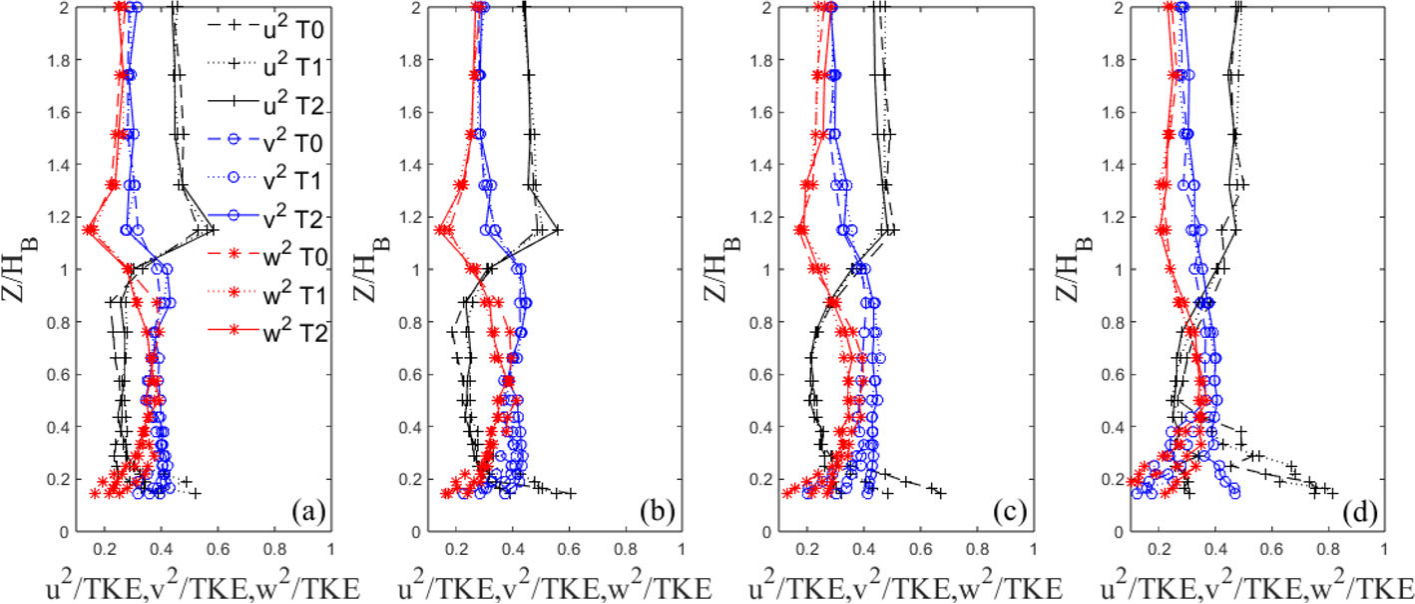
Vertical profiles of velocity fluctuations at four downstream locations ($X/H_{B}$=) **a** 0.39, **b** 0.64, **c** 1.12, **d** 2.05. T’ is used for Tall to make the figure more readable

**Fig. 19:**
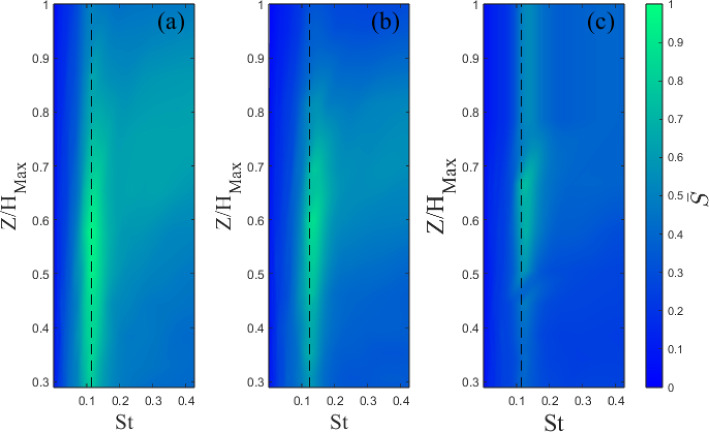
Wavelet magnitude at $X/H_{B}=0.67$ for Standard **a** 0. **b** 1, and **c** 2. Dashed line indicates the identified shedding frequency

**Fig. 20:**
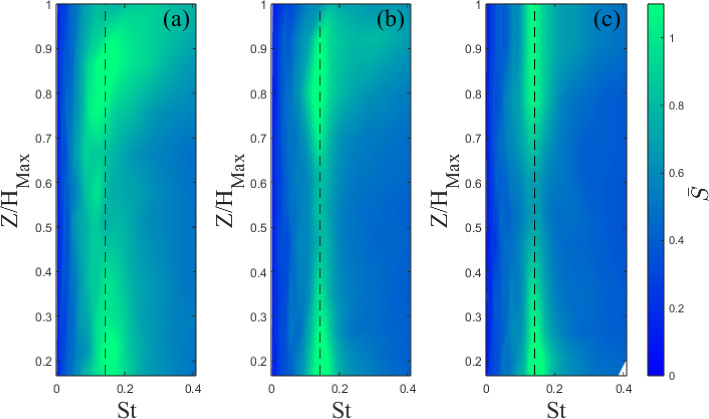
Wavelet magnitude at $X/H_{B}=0.67$ for Tall **a** 0 **b** 1, and **c** 2. Dashed line indicates the identified shedding frequency

Indeed, now that the overall TKE behaviour has been discussed, the contribution to the total TKE from each fluctuating velocity component is presented in Figs. 17 and 18, for the Standard and Tall models, respectively. It is clear to see from Fig. 17 that below the building height, $Z/H_B<1$, the $v'$ and $w'$ components make up the majority of the total contribution to the TKE of the Standard models at the first three streamwise locations (see Fig. 15a,b,c). The lateral fluctuations are dominant comprising nearly 50% of the total TKE at the nearest wall-normal stations to the wall at $X/H_B=1.94$. Here, large differences in the strength of the fluctuations are seen between each iteration. A similar picture can be drawn for the Tall models in Fig. 18. For all models, the $v'$ component provides the largest contribution to the total TKE followed by $w'$ (Figs. 17 and 18). The former is likely due to the strong spanwise shedding seen from both models (see Sect. 3.2.5, Figs. 19 and 20), while the latter is owing to the strong regions of recirculation present downstream of the models (Fig. 10). Around the roof level, the situation is reversed both for the Standard and Tall models (Figs. 17 and 18), with the streamwise fluctuations becoming the dominant contributors to the TKE owning to the small lateral and vertical turbulent motion in the freestream flow. It is also interesting to see that there are more differences across the strength of the fluctuation components in the case of the Tall buildings (see Fig. 18); this is in line with more three-dimensional flow characterising the higher aspect ratio models. Also of note is the concurrence of the profiles closer to the ground that is much worse for the Tall models in Fig. 18 than for the Standard buildings in Fig. 17. This suggests that these Tall models when arranged closely together, as in the case of a dense urban array, might generate larger dispersive stresses.

#### Shedding Frequency and Spectral Analysis

Wavelet analysis is used to investigate the distribution of turbulence energy intensity as a function of frequency and time. This was chosen over more conventional Fourier analysis, which is customarily used, as the signal time series are relatively short, while the flow is unsteady and as a result, wavelet analysis is deemed more effective in highlighting the shedding. Nguyen et al. (2023) also found this approach to be more effective at visualizing flow shedding when looking at tall buildings in isolation and clusters due to the inherent unsteadiness in the flow. The wavelet transformation of a time series is (Olhede and Walden (2002)):

4 $$\begin{aligned} S(a,b)=\frac{1}{\sqrt{a}}\int _{-\infty }^\infty \Psi ^*(\frac{t-b}{a})\xi (t)d(t), \end{aligned}$$

where *S* is the wavelet coefficient, $\Psi$ is the mother wavelet with $^*$ denoting the complex conjugate, *a* and *b* the scale and transition parameter, respectively. Here, we employ the Morse wavelet function (Olhede and Walden 2002). To give a better indication of the energetic scales, the mean wavelet magnitude ($\bar{S}$) is used. The $v'$ component is considered as it gives the clearest indication of the shedding, as it captures the signature of the spanwise vortices shed from the buildings.

Analysis is presented in Fig. 19 for the Standard cases and in Fig. 20 for the Tall building models. The shedding frequency, indicated by sharp peaks (lighter areas in Figs. 19 and 20), remains fixed with fractal iteration. The corresponding Strouhal number ($St=fW_B/U_{ref}$) is $St=0.12$ for the Standard ($St=0.14$ Tall) building models. These are in good agreement with previous studies (Nedić et al. 2013; Diaz-Daniel et al. 2017; Nguyen et al. 2023).

While the shedding frequency remains largely the same, its intensity is observed to decrease with each fractal iteration, most noticeably for the Standard models (attenuation of lighter areas, Fig. 19a–c). A decrease in intensity is also present for the Tall models but the changes are less visible (Fig. 20, colour bar. This corroborates s Nedić et al.’s (2013) findings of the same phenomenon using fractal flat plates.

Overall, this suggests the Strouhal number is largely independent of the model shape, but the additional length scales and geometric discontinuities reduce the intensity of the shedding, presumably as these act to break down the coherence of the wake’s turbulent structures and introduce 3D vorticity into a notionally 2D process. This is also consistent with larger velocity fluctuations observed in the base iteration models in the near-wake region (Fig. 15). As expected, the Tall models generally show a clearer and stronger region of shedding. This is likely due to the increased aspect ratio and the larger vertical separation between the horseshoe vortex system at the base of the model and the tip vortices. For the Standard models, their interaction can suppress the spanwise shedding (Wang et al. 2004), resulting in a less visible signature of the latter on the wavelet analysis. It is also striking to see that the most energetic frequencies are somewhat modulated by the changing geometry of the Tall models as a function of the wall-normal direction (Fig. 20), which is a less pronounced process for the Standard cases (Fig. 19).

**Fig. 21:**
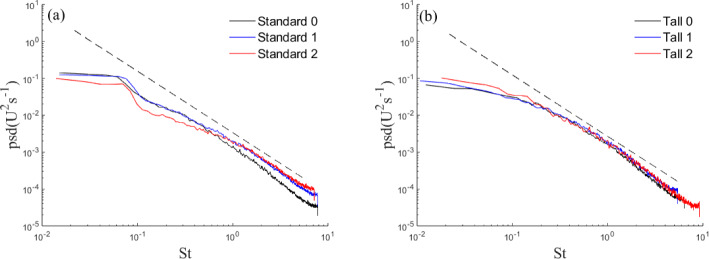
Velocity spectra of Standard (**a**) and Tall (**b**) building models taken at model mid-height and centre line for $X = 35$mm. The dashed line indicates the -5/3 line

Finally, to further aid the discussion in Sect. 3.1.1, we continue the frequency analysis by plotting the spectra of the streamwise velocity in Fig. 21 for a single point within the wake flow to asses if any redistribution in energy scales is apparent due to the introduction of progressively smaller scales in the building models. It is clear to observe, particularly for Standard 2 in Fig. 21a, a redistribution in the energy across scales with a reduction of energy in the low frequencies (i.e. large scales) and an increase in the higher frequencies (i.e. small scales). This process is less pronounced for the tall models in Fig. 21b, probably due to the full fractal pattern being limited to the top of the building, as previously discussed. These findings align well with one of the hypotheses discussed in Sect. 3.1.1, where the energy redistribution towards the dissipative range was hypothesised as one of the factors leading to a reduction in drag for the S2 model. However, from the wake recovery results (in Sect. 3.2.3), the Standard 2 model has the most persistent wake suggesting, as previously highlighted, that there is more at play here. To note that the wake recovery is based on a particular (*x*, *z*) along the centreline of the buildings (a 2D measurement), while the drag is an integral measurement (3D). Therefore, the behaviour of the drag for the standard models warrants further investigation.

## Conclusions

Wind tunnel experiments undertaken in the University of Surrey ‘Aero’ tunnel using Tall ($AR=3$) and Standard building models ($AR=1$) are analysed. From each, two additional fractal iterations are generated with a Minkowski sausage-type fractal generator to have minimum length scales roughly an order of magnitude less than the previous one while keeping the same mean width and height. All six models are immersed in a deep turbulent boundary layer giving a $H_{B}/\delta$ of 0.41 (Tall) and 0.24 (Standard).

The model drag measured with a FEFB increases significantly between iterations for Tall buildings by 8% (1) and 13% (2) relative to the base or iteration 0 model. For the Standard models, iteration 1 causes an increase of 5% but iteration 2 causes a decrease of 5% from the base model. An independent estimate of the drag is done using embedded static pressure ports in the 3D-printed models on the front and rear faces. Results are within 7% across cases suggesting the drag on large arrays of buildings could be acceptably evaluated via pressure measurements. This accuracy is similar to that in the log-law fitting of the rough-wall boundary layer via the modified Clauser chart method (Cheng and Castro (2002)). However, the spatial resolution/distribution of pressure ports needs to be prohibitively high to capture fully small changes seen across iterations. Nevertheless, smaller length scales did have a non-negligible effect on the drag.

The vertical and lateral wake spreads behind each building model are examined via 3D LDA measurements. For both sets of models, a linear lateral spread with the downstream location occurs linked to the areal extent of strong recirculation. For the Standard model, the base case has the largest lateral spread, whilst the opposite is true for the Tall models. Vertically, any significant wake spread is hindered by the roof-level shear layer growth. Vertical spread seems to scale with maximum building height, providing a good collapse of the data. For all three flow directions, the velocity fluctuations of the base iteration model are observed to be higher in the near-wake but to decay faster. Introducing progressively smaller scales does not modify the shedding frequency, but impacts its strength with the intensity of the shedding decreasing with each iteration.

This work explores the impact of additional length scales on isolated buildings’ drag and wake characteristics. Future work is required to verify whether the changes identified are still present –and significant– in large building arrays. This is relevant to urban flow and modelling as morphometric methods (Raupach 1992; Bottema 1993; Macdonald et al. 1998; Kanda et al. 2013) and canopy models (Coceal and Belcher 2004) typically do not capture length scale information. This work shows that this information might need to be included in these widely used frameworks.

## Data Availability

Most of the data in this manuscript are available online at the following repository: DOI 10.15126/surreydata.901481. Due to their sizes, time traces can be made available on request.
